# CB13, a novel PPARγ ligand, overcomes radio-resistance via ROS generation and ER stress in human non-small cell lung cancer

**DOI:** 10.1038/s41419-020-03065-w

**Published:** 2020-10-13

**Authors:** Tae Woo Kim, Da-Won Hong, Sung Hee Hong

**Affiliations:** Division of Radiation Biomedical Research, Korea Institute of Radiological and Medical Sciences, Seoul, 139-706 Korea

**Keywords:** Radiotherapy, Drug development

## Abstract

Peroxisome proliferator-activated receptor gamma (PPARγ) is a well-known therapeutic target for type 2 diabetes as well as is a potential target for effective anti-cancer drug, since PPARγ ligands such as ciglitazone (Cig) frequently cause cell death in many types of cancer cells and suppress tumor growth. However, many cancer patients acquire chemo-resistance or radio-resistance after chemo or radiotherapy, and it is still unclear. In the difficulty of well-known anti-cancer drugs, we developed a novel PPARγ agonist CB13 (1-benzyl-5-(4-methylphenyl) pyrido [2,3-d]pyrimidine-2,4(1H,3H)-dione) and investigated the anti-cancer effect and cell death mechanism on human non-small cell lung cancer (NSCLC) cells. With anti-cancer effect of Cig, CB13 also causes inhibition of cell growth by decreasing cell viability, increasing the release of LDH, and increasing caspase-3, and caspase-9 activities. CB13 generates reactive oxygen species (ROS) and causes cell death via ER stress in NSCLC and radio-resistant NSCLC cells (A549R and H460R), and a combination of CB13 and radiation induces greater ER stress and cell death when compared to CB13 alone. Taken together, our results suggest that a combination of CB13 and radiation may overcome radio-resistance caused by radiotherapy.

## Introduction

Lung cancer is a leading cause of cancer-related deaths and the most common cancer worldwide according to 2018 cancer statistics^1^. Lung cancers are classified into two types: small-cell lung cancer (SCLC) and non-small-cell lung cancer (NSCLC). SCLC is responsible for ~10–15% of all lung cancer cases, and NSCLC is responsible for the other 80–85%^2^. NSCLC is comprised of more epithelial lung cancer types than SCLC, and it frequently becomes resistant to chemotherapy^3^. Radio therapy is an effective therapeutic strategy against lung cancer, but it frequently causes radio-resistance^4^. Therefore, understanding the mechanism that cause radio-resistance is important for maintaining radiotherapy efficacy. Radio-resistance is caused by clonogenic cells within the tumor and by acquired cancer radio-resistance^5,6^. Radiation often causes an EMT phenotype, and cancer cells that gain an EMT phenotype present a large obstacle for effective radiotherapy^7^. Therefore, when developing novel and effective drugs for the treatment of lung cancer, it is important to focus on overcoming radio-resistance. Peroxisome proliferator-activated receptors (PPARs) are type II nuclear hormone receptors for thyroid hormone, retinoic acid, and steroid^8^. PPARs form heterodimers with retinoic X receptor (RXR) and become activated by specifically binding to PPAR response elements (PPREs)^9,10^. There are three types of PPARs: α, β, and ɣ. Each is encoded by a separate gene and displays a distinct pattern of tissue distribution^11,12^. PPARɣ has anti-inflammatory properties and plays a central role in the differentiation of adipocyte cells, tumor suppression, and metabolism^13,14^. PPARɣ is up-regulated in many cancer cell types, but the effect of numerous PPARɣ ligands, including 15-deoxy-Δ12,14-prostaglandin J2 and thiazolidinediones (TZDs), inhibit tumor growth in several cancer types (e.g. lung, ovarian, pancreatic, and colon) and cause cell death^15–20^. Thus, these receptors are an attractive target and have made a significant impact on novel synthetic anti-cancer agent development. An increasing number of studies have demonstrated that TZD, a synthetic class of PPARɣ ligand that includes ciglitazone (Cig), rosiglitazone, pioglitazone, and troglitazone, prevents cancer cell growth^21,22^. Recent reports have shown that PPARɣ receptors and their ligands can suppress tumor growth by mediating cell cycle arrest and apoptosis in colon cancer^23^. One recent study suggested that PPARɣ ligands cause cell death in various cancers and regulate diverse cancer pathways^24^.

The endoplasmic reticulum (ER) participates in various functions, including protein folding, post-translational modification, secretory protein synthesis, and cellular calcium storage. In addition to these functions, the unfolded protein response (UPR) is a cellular stress response that causes cellular damage and apoptosis^25^. UPR is mediated by three membrane transducers: pancreatic ER kinase-like endoplasmic reticulum kinase (PERK), activating transcription factor (ATF6), and inositol requiring enzyme 1 (IRE1)^26^. UPR is a cellular protective response against ER stress conditions, but an accumulation of UPR initiates pro-apoptotic signaling that includes the activation of CHOP (also known as GADD153)^27^. Many studies have shown that TZDs can up-regulate ER stress markers and cause cell death in breast cancer cells. In breast cancer cells, Δ2-troglitazone triggers cell death via ER stress^28^. Both Cig and troglitazone activate the PERK-elF2α pathway in rat liver epithelial cells^29^. Cig, a known PPARɣ ligand, induces ER stress-mediated cell death via the PERK axis in NSCLC cells and contributes to caspase-3, -4, -8, -9, and -12 cleavage^30^. Mounting evidence indicates that ROS production leads to ER stress-induced cell death, and ER stress is also regulated by ROS inhibitors, which indicates ROS is a regulator of ER stress^31^. An increasing number of reports have shown that exosomes, nanoscale vesicles (50–150 nm in diameter), participate in cell–cell communication and confer ER stress signals^32^. Particularly, we found a relationship between PPARɣ ligands and exosomes in NSCLC cells. These novel finding elaborates on the anti-cancer mechanism that allow CB13 treatment to overcome radio-resistance.

In this study, we evaluated the anti-cancer effect of CB13 (a novel PPARγ agonist candidate) and investigated the role of PPARɣ, ROS, exosomes, and ER stress in CB13-induced cell death in human NSCLC and radio-resistant NSCLC cells. Moreover, we demonstrated that CB13 and radiation co-treatment can overcome radio-resistance via ER stress in radio-resistant NSCLC cells.

## Results

### Identification of CB13 as a novel PPARɣ ligand in 3T3-L1 cells

**Fig. 1:**
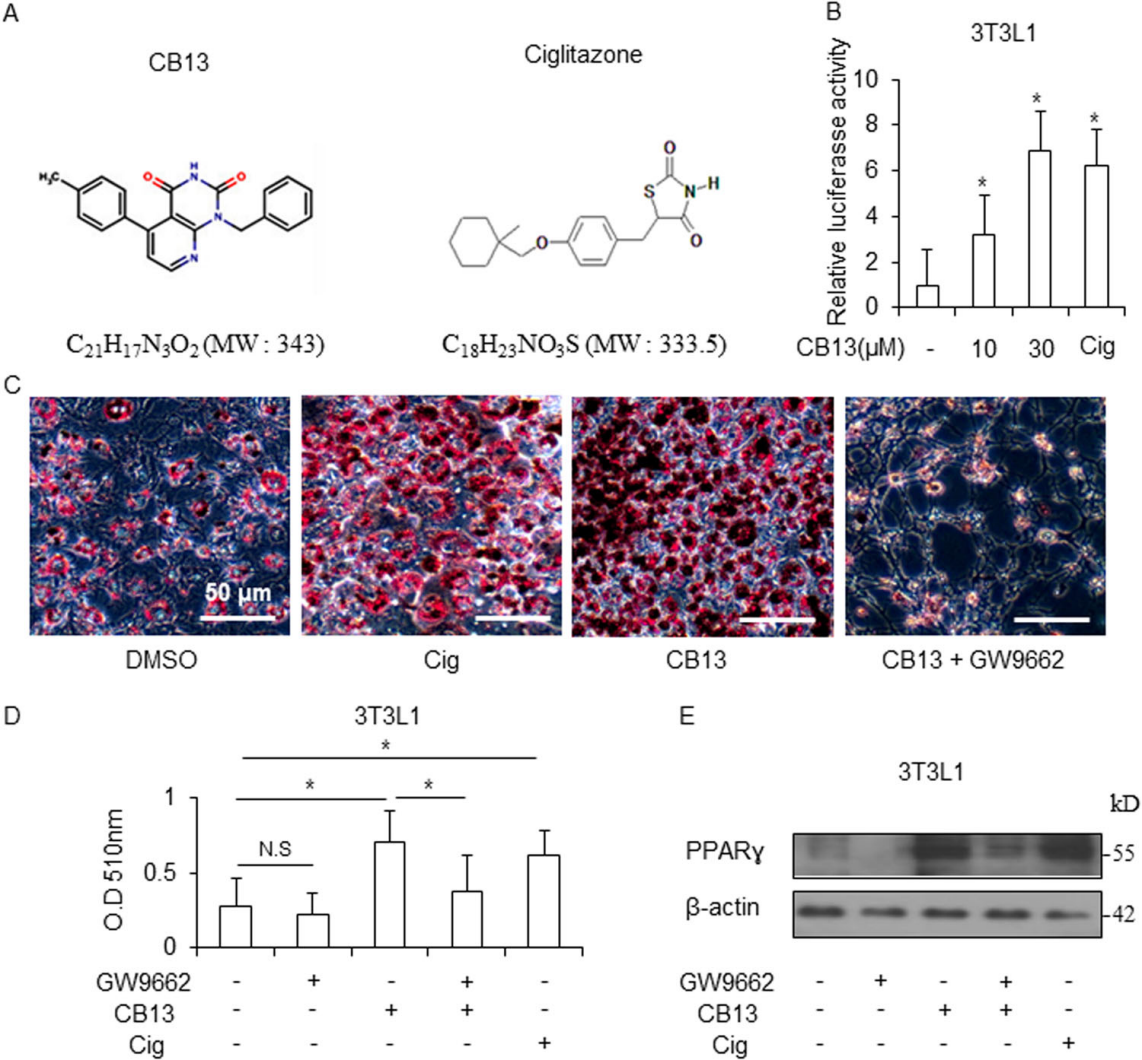
Adipocyte differentiation in CB13-treated 3T3-L1 cells. **a** The chemical structures and molecular formulas of CB13 and Cig. **b** 3T3-L1 cells were transiently transfected with 2 μg of the PPAR response element reporter gene (pGL3-PPRE vector) and the pGL3-PPRE vector and treated with CB13 or Cig at the indicated doses (CB13, 10 and 30 μΜ; Cig, 10 μΜ); *P < 0.05. **c** CB13-mediated (30 μΜ, 24 h), Cig-mediated (10 μΜ, 24 h), or CB13/GW9662-mediated adipocyte differentiation was assessed by the presence of Oil Red O-stained droplets. Oil Red O-stained cells were detected using a light microscope and scoring cells from each dish at ×400 magnification. **d** Oil Red O quantification was performed by adding a dye extraction solution to each well and measuring the absorbance at 510 nm. Values indicate the means ± SE of three replicates (*P < 0.05, **P < 0.01 vs. control; Student’s *t*-test). **e** Effect of GW9662 on CB13-treated 3T3-L1 cells. 3T3-L1 cells were pretreated with GW9662 (20 μΜ) for 4 h and then treated with CB13 (30 μΜ). 3T3-L1 cells were also treated with Cig (10 μΜ) as a control. Total lysates were interrogated by Western blot analysis to confirm inhibition of PPARɣ. β-actin was used as a protein loading control.

In an effort to identify novel anti-cancer agents, we obtained (1-benzyl-5-(4-methylphenyl) pyrido [2,3-d] pyrimidine-2,4(1H,3H)-dione) (CB13) from ChemBridge. Figure 1a shows the chemical structures of CB13 and Cig. PPARɣ ligands, including Cig and rosiglitazone, frequently induce adipocyte differentiation and play a key role in regulating adipogenesis^33^. To confirm CB13’s capacity as a PPARɣ ligand, we measured PPRE-luciferase activity in 3T3-L1 cells exposed to CB13 and Cig. Both CB13 and Cig increased luciferase activity in 3T3-L1 cells (Fig. 1b). We determined whether CB13, as a PPARɣ ligand candidate, could differentiate adipocytes in 3T3-L1 cells using Cig as a positive control. As shown in Fig. 1c and d, Oil Red O staining confirms that CB13 and Cig cause adipocyte differentiation in 3T3-L1 cells, and GW9662, a PPAR-γ antagonist, blocks CB13-induced adipocyte differentiation. Western blot analysis revealed that CB13 and Cig mediated increased PPARɣ expression, but GW9662 suppressed CB13-induced PPARɣ expression (Fig. 1e).

### CB13 induces cell death in NSCLC cells

**Fig. 2:**
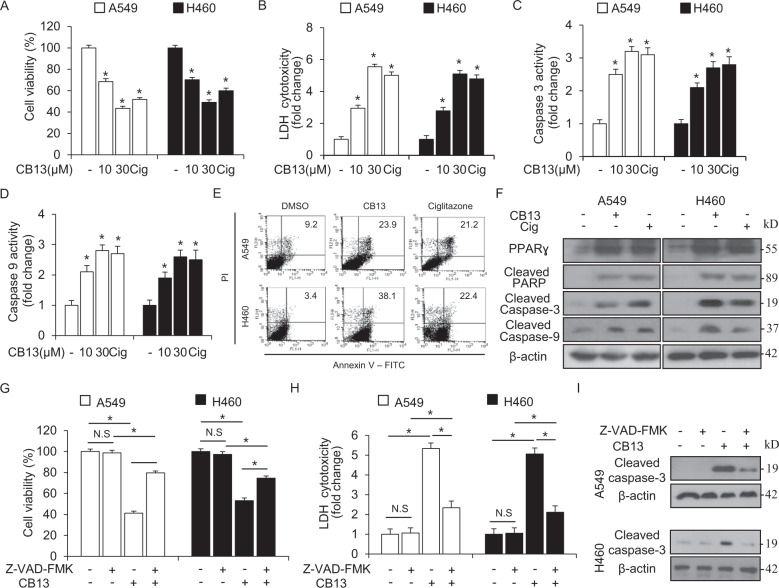
CB13 causes apoptotic cell death in NSCLC cells. **a**–**d** A549 and H460 cells were treated with CB13 or Cig at the indicated doses (CB13, 10 and 30 μΜ; Cig, 10 μΜ). Cell viability and LDH assays were performed and caspase-3 and -9 activities were monitored under these conditions; *P < 0.05. **e** Flow cytometric analysis of Annexin-V-FITC and propidium iodide staining in CB13-treated (30 μM, 24 h) and Cig-treated (10 μM, 24 h) A549 and H460 cells. **f** Western blot analyses of PPARɣ, cleaved PARP, and cleaved caspase-3 and -9 at the indicated doses in CB13-treated (30 μM, 24 h) and Cig-treated (10 μM, 24 h) A549 and H460 cells. β-actin was used as a protein loading control. **g**–**i** A549 and H460 cells were pretreated with Z-VAD-FMK (50 μΜ) for 4 h and then treated with CB13 (30 μΜ, 24 h). Cell viability and cell cytotoxicity were determined using WST-1 and LDH assays; *P < 0.05. Cleaved caspase 3 expression levels in the protein samples were determined via Western blot analyses. β-actin was used as a protein loading control.

To investigate the cytotoxic effect of CB13, WST-1, and LDH assays were performed and caspase-3 and -9 activities were used to monitor the survival of A549 and H460 cells after treatment with CB13 or Cig. CB13 and Cig reduced cell viability, increased LDH release, and increased caspase-3 and -9 activities (Fig. 2a–d). An annexin V FACs assay showed an increase in apoptosis signaling in CB13- and Cig-treated A549 and H460 cells (Fig. 2e). To examine the molecular mechanisms of CB13-mediated cell death, Western blot analyses were performed. They indicate that CB13 and Cig increased PARP cleavage, caspase-3 levels, and caspase-9 levels in A549 and H460 cells (Fig. 2f). These results demonstrate that CB13 mediates anti-cytotoxic effects by inducing apoptosis in NSCLC cells. In a pharmacological experiment using the caspase inhibitor, Z-VAD-FMK, we co-treated A549 and H460 cells with Z-VAD-FMK and CB13. This combination prevented CB13 from decreasing cell viability and increasing LDH release as well as caspase-3 and -9 cleavage (Fig. 2g–i).

### CB13 induces cell death via ER stress in NSCLC cells

**Fig. 3:**
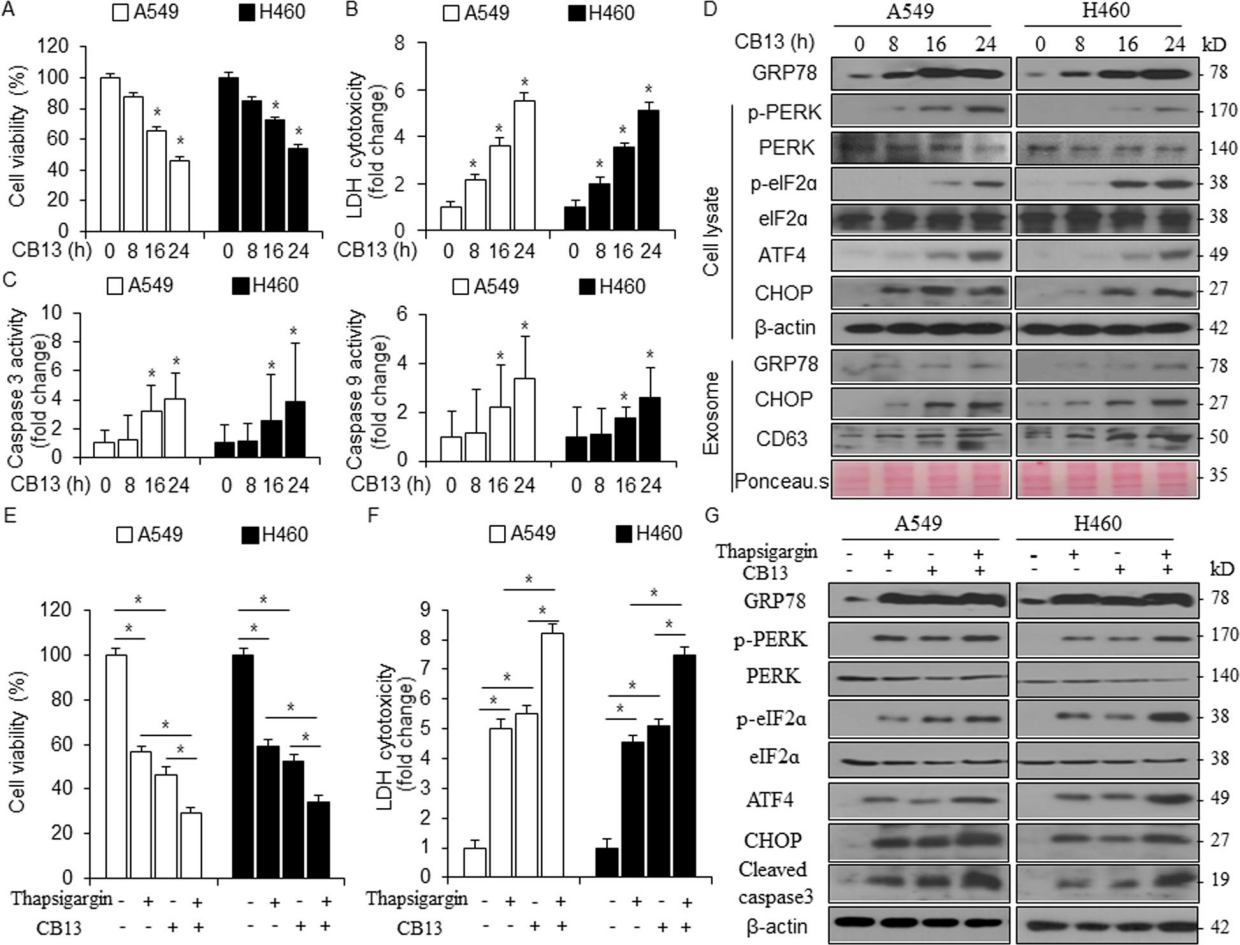
CB13 causes apoptotic cell death via ER stress in NSCLC cells. **a**–**c** A549 and H460 cells were incubated with CB13 (30 μM) for various amounts of time (0, 8, 16, and 24 h) of CB13 (30 μM), and WST-1, LDH, and caspase-3 and -9 activity assays were performed under these conditions; *P < 0.05. **d** GRP78, p-PERK, p-eIF2α, eIF2α, ATF4, CHOP, and CD63 expression levels in the protein samples isolated from cells and exosomes were determined via Western blot analyses. β-actin and Ponceau S stain were used as a protein loading control. **e**–**g** A549 and H460 cells were treated with thapsigargin (3 μΜ, 24 h) and CB13 (30 μΜ, 24 h). Cell viability and LDH release were determined using WST-1 and LDH assays, respectively; *P < 0.05. GRP78, p-PERK, p-eIF2α, eIF2α, ATF4, CHOP, and cleaved caspase-3 expression levels in the protein samples were determined via Western blot analyses. β-actin was used as a protein loading control.

To further determine the mechanism through which CB13 regulates cell death, we treated A549 and H460 cells with CB13 in a time-dependent manner and performed WST-1, LDH, and caspase-3 and -9 activity assays. The results indicate that CB13 reduced cell viability, increased LDH cytotoxicity, and increased caspase-3 and -9 activities in a time-dependent manner (Fig. 3a–c). A recent report suggested that ER stress plays an important role in cell death, and CHOP is a major component of the ER stress-mediated apoptosis pathway^34^. To investigate whether CB13 causes ER stress-mediated cell death, we treated A549 and H460 cells with CB13 in a time-dependent manner and measured various ER stress signals using Western blot analysis. From the cell lysates generated in this experiment, we observed increased GRP78, p-PERK, p-eIF2α, ATF4, and CHOP expression (Fig. 3d). Interestingly, in this condition, we isolated exosomes from the supernatants and quantified them using Ponceau S staining and immunoblotting with a CD63-specific antibody (Fig. 3d). The Western blot analysis suggests that exosomes mediate time-dependent ER stress-induced cell death in cells treated with CB13, which indicates the induction of GRP78 and CHOP (Fig. 3d). These results suggest that ER stress confers cell death via PERK-eIF2α-ATF4-CHOP signaling in CB13-treated cells. Pharmacological studies revealed a decrease in cell viability and an increase in LDH cytotoxicity after treatment with thapsigargin (TG, an ER stress inducer) or CB13, which indicates cell death. Furthermore, A549 and H460 cells treated with a combination of TG and CB13 had lower cell viability and a greater increase in LDH release than A549 and H460 cells treated with CB13 or TG, alone (Fig. 3e, f). Under these conditions, Western blot analysis indicated that cells co-treated with TG/CB13 showed higher levels of GRP78, p-PERK, p-eIF2α, ATF4, and CHOP expression and higher levels of caspase-3 cleavage than cells treated with CB13 or TG, alone (Fig. 3g).

### Inhibition of ER stress suppresses CB13-induced cell death in NSCLC cells

**Fig. 4:**
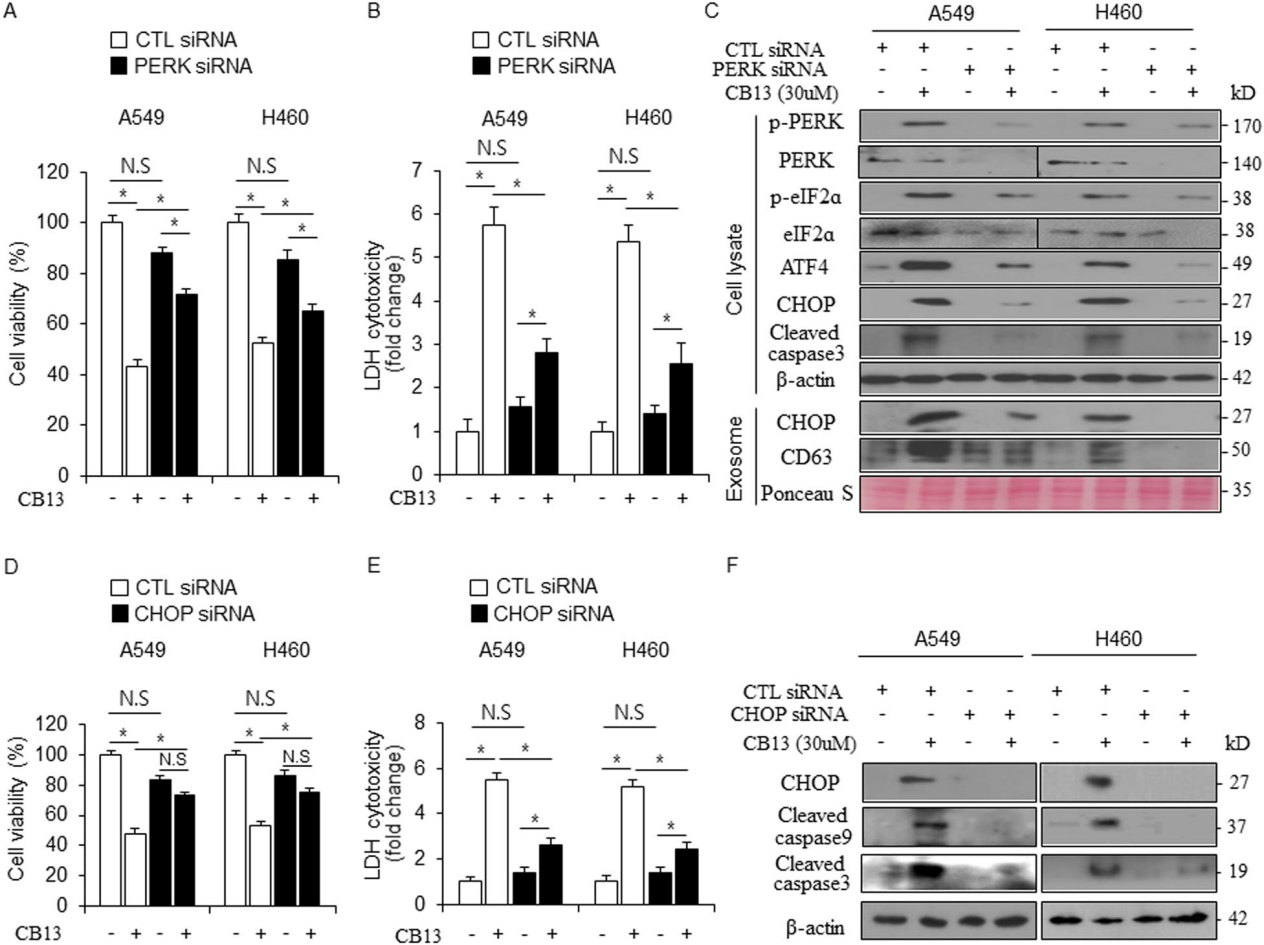
Inhibition of ER stress blocks CB13-induced apoptotic cell death in NSCLC cells. **a**–**c** After A549 and H460 cells were transfected with PERK siRNAs, cell viability assays, LDH assays, and Western blot analyses were performed to examine p-PERK, p-eIF2ɑ, ATF4, CHOP, and cleaved caspase-3 levels after CB13 (30 μΜ, 24 h) treatment; *P < 0.05. β-actin was used as the protein loading control. After exosome isolation, a Western blot analysis was also performed to examine CHOP and CD63 levels. β-actin and Ponceau S stain were used as a protein loading control. **d**–**f** After A549 and H460 cells were transfected with CHOP siRNAs, cell viability assays, LDH assays, and Western blot analyses were performed to examine CHOP, cleaved caspase-9, and cleaved caspase-3 levels after CB13 (30 μΜ, 24 h) treatment; *P < 0.05. β-actin was used as the protein loading control.

To further investigate whether CB13 regulates cell death through ER stress in NSCLC cells, we treated A549 and H460 cells with CB13 after knocking down PERK and CHOP with specific siRNAs. CB13 did not decrease cell viability, and it did not increase LDH release in PERK or CHOP knockdowns when compared to control (CTL) cells (Fig. 4a, b, d, e). Western blot analysis suggested that CB13 suppresses PERK and eIF2α phosphorylation, caspase-3 cleavage, and ATF4 and CHOP expression in PERK knockdown cells when compared to CTL cells (Fig. 4c). Moreover, when exosome samples were stained with Ponceau S and quantified, they indicated that CB13 inhibits CHOP and CD63 expression in PERK knockdown cells when compared to CTL cells (Fig. 4c). Western blot analysis revealed that CB13 blocks CHOP expression and caspase-3 and -9 cleavage in CHOP knockdown cells when compared to CTL cells (Fig. 4f).

### CB13 induces ER stress and cell death via ROS generation in NSCLC cells

**Fig. 5:**
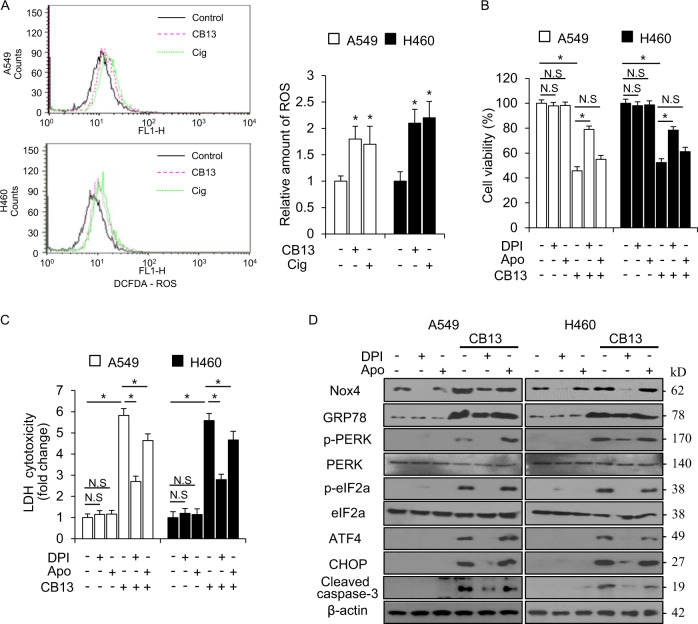
DPI inhibits CB13-mediated apoptotic cell death in NSCLC cells. **a** FACs data indicate the change in fluorescence intensity of DCFDA dye in CB13-treated (30 μΜ, 8 h) or Cig-treated (10 μΜ, 24 h) A549 and H460 cells; *P < 0.05. **b**–**d** A549 and H460 cells were treated with DPI (1 μM, 24 h), Apo (100 μM, 24 h), and CB13 (30 μΜ, 24 h). Cell viability was determined using a WST-1 assay, and LDH cytotoxicity was measured using an LDH assay; *P < 0.05. A Western blot analysis examining GRP78, p-PERK, p-eIF2ɑ, ATF4, CHOP, and cleaved caspase-3 levels was performed using these samples. β-actin was used as the protein loading control.

An increasing number of studies suggest that ER stress and the generation of reactive oxygen species (ROS) are closely related, and the related pathway may regulate cell death^35^. Elevated ROS production induces cell death via ER stress in cancer cells, and ROS scavengers (e.g. NAC) regulate ER stress and cell death through ROS generation, indicating ROS drives ER stress^36^. To further investigate whether CB13 mediates ROS generation, we examined intracellular ROS with flow cytometry after labeling with the fluorescent probe DCFDA. After an 8 h incubation with CB13, DCFDA fluorescence increased by 1.5–2-fold in A549 and H460 cells when compared to DMSO-treated cells (Fig. 5a). To probe the sources of CB13-mediated ROS production in A549 and H460 cells, several ROS inhibitors such as Apo and DPI were used in combination with CB13. As shown in Fig. 5b, c, DPI dramatically inhibited CB13’s ability to reduce cell viability and increase LDH cytotoxicity, but not Apo. Western blot analysis revealed that DPI inhibits CB13 from increasing the expression of GRP78, p-PERK, p-eIF2α, ATF4, CHOP, and caspase-3 cleavage in A549 and H460 cells, but not Apo (Fig. 5d).

### PPARɣ inhibition suppresses CB13-mediated cell death in NSCLC cells

**Fig. 6:**
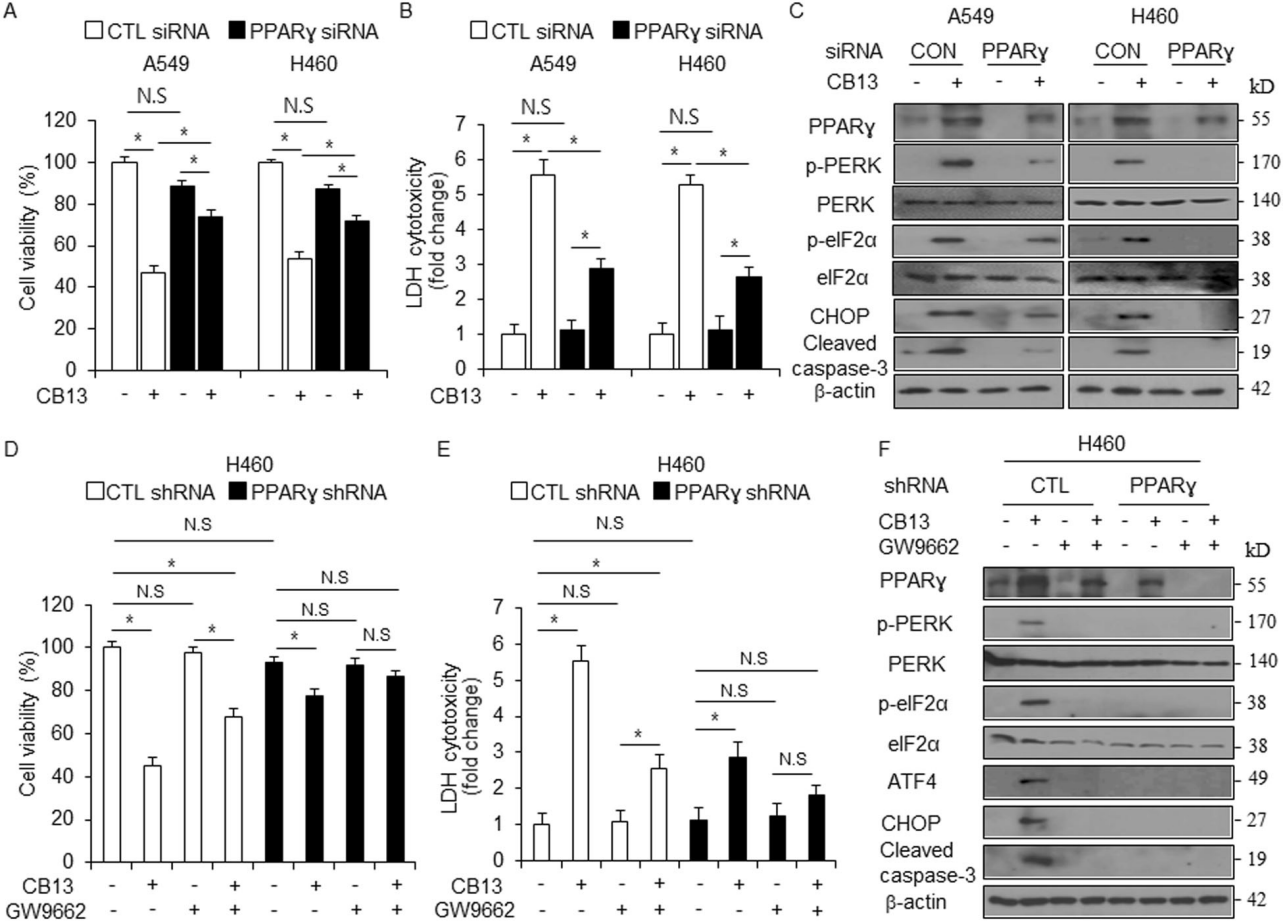
Inhibition of PPARɣ blocks CB13-mediated apoptotic cell death in NSCLC cells. **a**–**c** A549 and H460 cells were transfected with PPARɣ siRNAs and treated with CB13 (30 μΜ, 24 h). Next, cell viability assays, LDH assays, and Western blot analyses were performed to examine the expression levels of PPARɣ, p-PERK, p-eIF2ɑ, CHOP, and cleaved caspase-3; *P < 0.05. β-actin was used as a protein loading control. **d**–**f** PPARɣ shRNA stable H460 cell lines were established after H460 cells were transfected with PPARɣ shRNA. These cells were treated with CB13 (30 μΜ, 24 h), GW9662 (20 μM, 24 h), or CB13/GW9662, and cell viability assays, LDH assays, and Western blot analyses were performed to examine PPARɣ, p-PERK, p-eIF2ɑ, ATF4, CHOP, and cleaved caspase-3 expression; *P < 0.05. β-actin was used as a protein loading control.

PPARɣ ligands, including 1-(*trans*-methylimino-N-oxy)-6-(2-morpholinoethoxy)-3-phenyl-(1H-indene-2-carboxylic acid ethyl ester) (KR-62980) and rosiglitazone, exert anti-lung cancer effects by inducing ROS production, apoptosis, and differentiation and by inhibiting cell viability^37^. To further test whether CB13 mediates cell death via PPARɣ activation, we performed knockdown experiments using PPARɣ-specific siRNAs in A549 and H460 cells, cell viability and LDH assays, and Western blot analyses. The results show that CB13 did not decrease cell viability, and it did not increase LDH cytotoxicity in PPARɣ knockdown cells when compared to CTL cells (Fig. 6a, b). After quantifying PPARɣ knockdown in A549 and H460 cells, Western blot analysis indicated that CB13 induces PPARɣ, p-PERK, p-eIF2α, and CHOP expression and cleavage of caspase-3 in cells transfected with CTL siRNAs. Conversely, CB13 inhibits the expression of these proteins in PPARɣ knockdown cells (Fig. 6c). To further investigate whether PPARɣ activation regulates CB13-mediated cell death in NSCLC cells, we transfected H460 cells with PPARɣ-specific shRNAs. Positive cells were selected with puromycin and established into stable PPARɣ knockdown cells. We treated PPARɣ knockdown stable H460 cells with CB13, GW9662, or CB13/GW9662, and performed cell viability and LDH assays as well as Western blot analyses. In CTL cells, GW9662 had no effect, CB13 decreased cell viability and increased LDH cytotoxicity, and GW9662/CB13 inhibited CB13’s ability to reduce cell viability and increase LDH release when compared to CB13 treatment, alone. However, in PPARɣ shRNA stable H460 cells, CB13, GW9662, and GW9662/CB13 had no effect on cell viability or LDH release (Fig. 6d, e). In CTL cells, Western blot analyses suggested that CB13 induces the expression of PPARɣ, p-PERK, p-eIF2α, ATF4, and CHOP and cleavage of caspase-3, but GW9662 and GW9662/CB13 blocks the expression of PPARɣ, p-PERK, p-eIF2α, ATF4, and CHOP and cleavage of caspase-3 (Fig. 6f). In PPARɣ shRNA stable H460 cells, CB13, GW9662, and GW9662/CB13 did not affect the expression of the above markers (Fig. 6f).

### A combination of CB13 and radiation overcomes radio-resistance

**Fig. 7:**
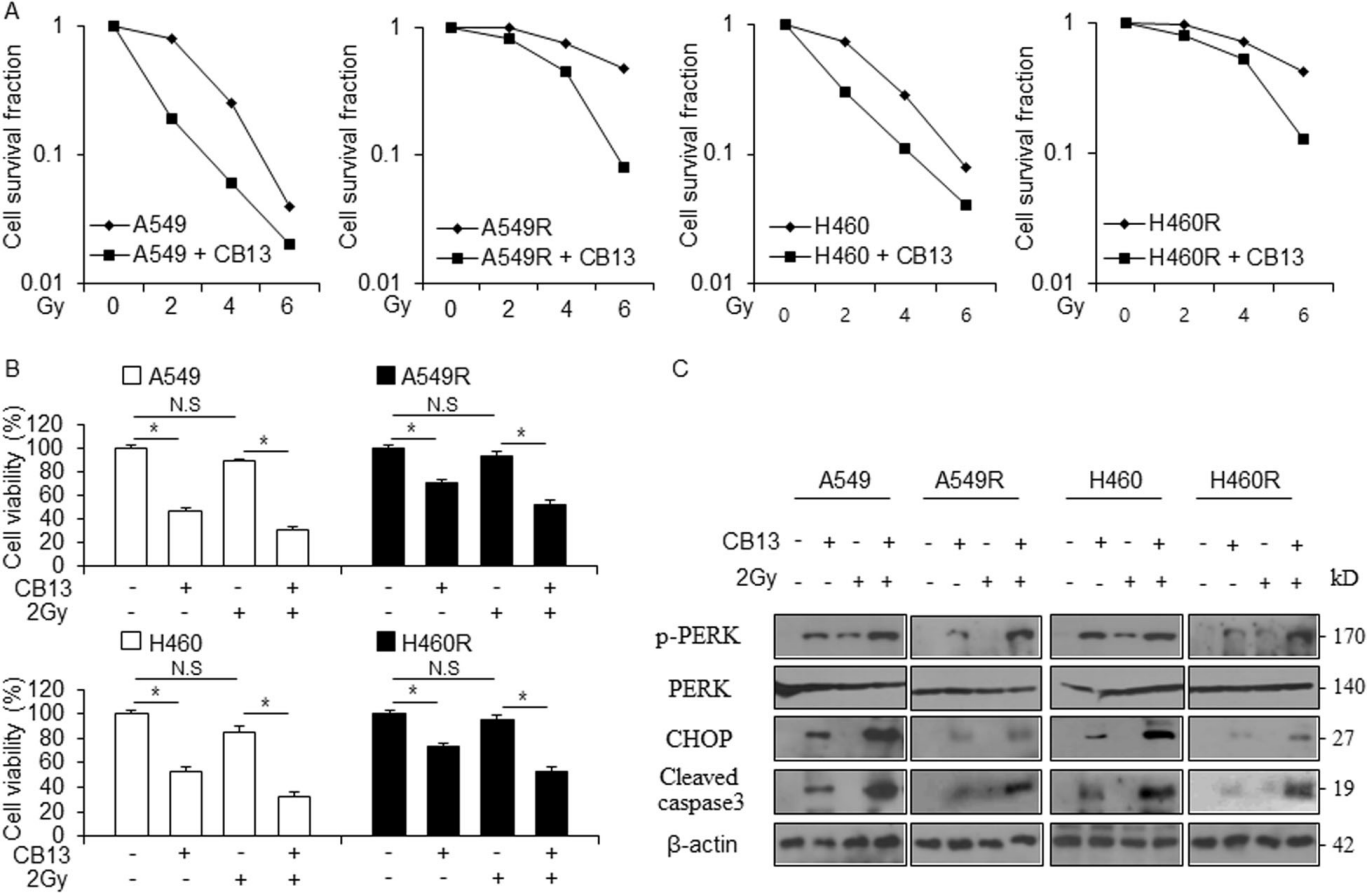
2 Gy/CB13 induces apoptotic cell death via ER stress in radio-resistant NSCLC cells. **a** A clonogenic cell survival assay was conducted with CB13 (30 μΜ, 24 h) treatment after exposure to various radiation doses (2, 4, or 6 Gy). The survival fraction was calculated using the surviving fraction formula in A549, H460, A549R, and H460R cells; *P < 0.05. **b** and **c** A549, H460, A549R, and H460R cells were treated with CB13 (30 μΜ, 24 h) after radiation exposure with 2 Gy. A cell viability assay was performed along with a Western blot analysis to examine p-PERK, CHOP, and cleaved caspase-3 expression.

Radiotherapy is a common therapeutic strategy for the treatment of NSCLC even though radio-resistance frequently occurs. Exactly how radio-resistance occurs is still unclear and better mechanistic studies are necessary to discover the underlying causes^38,39^. To assess whether CB13 causes radio-sensitivity in radio-resistant NSCLC cells, we performed colony formation assays, cell viability assays, and Western blot analyses. Our results showed that CB13 causes a synergistic reduction in the surviving fraction levels depending on the level of radiation exposure (2, 4, or 6 Gy) on NSCLC (A549 and H460) and radio-resistant NSCLC cells (A549R and H460R) when compared to control cells (Fig. 7a). Additionally, surviving fraction levels were higher in CB13-treated A549 and H460 cells when compared to CB13-treated A549R and H460R cells (Fig. 7a). In A549 and H460 cells, CB13 decreased cell viability, 2 Gy/CB13 decreased cell viability even further, and 2 Gy, alone, had no effect (Fig. 7b). In A549R and H460R cells, CB13 decreased cell viability, 2 Gy/CB13 decreased cell viability even further, and 2 Gy, alone, had no effect (Fig. 7b). Furthermore, CB13 or 2 Gy/CB13 causes greater cell viability in A549R and H460R cells when compared to A549 and H460 cells (Fig. 7b). To probe whether 2 Gy/CB13 contributes to cell death via ER stress, we carried out a Western blot analysis. In A549 and H460 cells, 2 Gy of radiation caused an increase in p-PERK expression, but it had no effect on the expression of CHOP or cleavage of caspase-3. CB13 treatment up-regulated the expression of p-PERK and CHOP and cleavage of caspase-3 in A549 and H460 cells when compared to 2 Gy treatment, alone. 2 Gy/CB13 treatment dramatically increased the expression of p-PERK and CHOP (ER stress markers) and cleavage of caspase-3 when compared to CB13 alone (Fig. 7c). In A549R and H460R cells, 2 Gy, alone, had no effect on the expression of most ER markers, and CB13 weakly increased the expression of p-PERK and CHOP and cleavage of caspase-3. Interestingly, 2 Gy/CB13 synergistically increased the expression of p-PERK and CHOP and the cleavage of caspase-3 in A549R and H460R cells.

### PPARɣ inhibition suppresses 2 Gy/CB13-induced radio-sensitivity

**Fig. 8:**
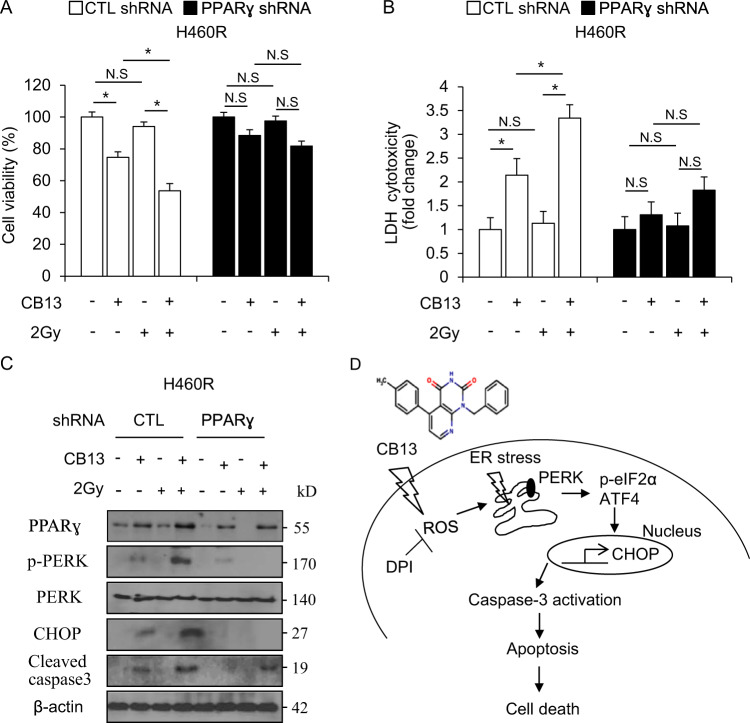
Inhibition of PPARɣ suppresses 2 Gy/CB13-induced apoptotic cell death in radio-resistant H460R cells. **a**–**c** After H460R cells were transfected with PPARɣ shRNA, they established PPARɣ shRNA stable H460R cell lines. These cells were treated with CB13 (30 μΜ, 24 h) after 2 Gy exposure, and cell viability assays, LDH assays, and Western blot analysis was performed to examine PPARɣ, p-PERK, CHOP, and cleaved caspase-3 expression; *P < 0.05. β-actin was used as a protein loading control. **d** Schematic representation of the apoptotic cell death pathways stimulated by CB13 in NSCLC and radio-resistant NSCLC cells.

To further investigate whether 2 Gy/CB13 can overcome radio-resistance, we established stable PPARɣ knockdown cells by transfecting PPARɣ shRNA in H460R cells. We treated these stable knockdown cells with CB13, 2 Gy, or 2 Gy/CB13 and performed cell viability assays, LDH assays, and Western blot analyses. In control (CTL) stable H460R cells, CB13 decreased cell viability and increased LDH cytotoxicity. CB13, in combination with 2 Gy, caused even lower cell viability and higher LDH release, but 2 Gy, alone, had no effect. However, in PPARɣ knockdown stable H460R cells, CB13, 2 Gy, and 2 Gy/CB13 had no effect, which indicates PPARɣ inhibition blocks 2 Gy/CB13-mediated radio-sensitivity of radio-resistant cells (Fig. 8a, b). Western blot analysis showed that 2 Gy did not induce the expression of the indicated markers in CTL stable H460R cells. CB13 induced weak expression of PPARɣ, p-PERK, and CHOP and cleavage of caspase-3, but 2 Gy/CB13 caused dramatically higher expression levels of PPARɣ, p-PERK, and CHOP and cleavage of caspase-3 when compared to 2 Gy or CB13, alone (Fig. 8c). In PPARɣ knockdown H460R cells, CB13 treatment caused a small increase in PPARɣ expression, and 2 Gy or 2 Gy/CB13 showed almost no increase in PPARɣ expression.

## Discussion

In this study, we showed that CB13, a novel PPAR-γ agonist, causes cell death via ROS production and ER stress in human NSCLC cells. Also, DPI, a ROS inhibitor, suppresses CB13-induced cell death. CB13, in combination with radiation (2 Gy), induces cell death via ER stress in radio-resistant NSCLC cells; however, inhibition of ER stress or PPARɣ blocks cell death in CB13-treated radio-resistant NSCLC cells. These results suggest that CB13 is a novel anti-tumor agent that overcomes radio-resistance in NSCLC.

PPARɣ is expressed in a variety of cancer types, including breast, colon, lung, liver, pancreas, stomach, neuroblastoma, and thyroid^40^. PPARɣ ligands such as 15D-PGJ_2_, TGZ, RGZ, Cig, pioglitazone, and rosiglitazone are used to treat diabetes, but they also inhibit the proliferation of cancer by influencing apoptosis pathways^41^. 15D-PGJ2 and Cig cause cell death via a PPARɣ-dependent pathway, but TZD and rosiglitazone cause cell death via both PPARɣ-dependent and PPARɣ-independent pathways, respectively^42,43^. Our findings suggest that CB13 exhibits powerful anti-cancer effects through PPARɣ expression in NSCLC cells, (similar to Cig), and knocking down PPARɣ inhibits CB13-induced PPARɣ expression and cell death. Thus, the apoptosis signaling axis through CB13-induced PPARɣ expression may be a potential pathway in anti-cancer therapy. PPARɣ ligands regulate caspase-dependent apoptosis pathways in various cancer types (e.g. lung, liver, breast, colon, and brain)^44^. CB13 and Cig cause cell death via caspase-9- and -3-dependent pathways in NSCLC cells, respectively. Conversely, Z-VAD-FMK, a caspase inhibitor, blocks CB13-induced caspase-dependent apoptosis in NSCLC cells. Taken together, CB13 treatment up-regulated apoptosis-related markers inhibited by Z-VAD-FMK, and there is a close relationship between CB13 treatment and apoptosis.

ROS plays roles in both cell survival and cell death in various cancer types^45^. Increased ROS production causes apoptosis and DNA damage in various cancers and has diverse anti-cancer effects^46^. 15D-PJ2 causes apoptosis and decreases cancer cell proliferation by increasing ROS production and proline oxidase (POX, mitochondrial redox enzyme) levels in cancer cells, and the inhibition of POX blocks PPARɣ-induced ROS generation^47^. Cig also induces cell death through ROS generation in NSCLC cells, and NAC suppresses Cig-induced apoptosis^48^. Our data suggest that Apo does not inhibit CB13-mediated ROS production or cell death in NSCLC cells, but DPI suppresses CB13-induced ROS generation and apoptosis. These findings indicate that CB13 causes cell death via ROS production in NSCLC cells and may exist with a variety of anti-oxidant defences, such as superoxide dismutases (SOD), catalase, glutathione peroxidase, peroxiredoxins, glutathione (GSH), lipoic acid, carotenoids, and iron chelators.

ROS regulates various cellular processes (e.g. signal transduction, DNA repair, and protein folding), and excessive ROS activates pro-apoptotic signaling pathways that involve mitochondrial dysfunction, ER stress, and DNA damage^49^. Recent reports suggest that PPARɣ ligands induce ER stress and apoptosis in NSCLC cells, and CHOP, a major mediator of ER stress, is a key marker of ER stress-induced cell death^50^. Furthermore, ER stress activates the UPR, which is regulated by trans-membrane receptors including PERK, ATF6, and IRE1α^51^. CB13 up-regulates ER stress markers (e.g. p-PERK, p-eIF2α, ATF4, cleaved caspase-3, and CHOP), and TG, an ER stress inducer, has a synergic effect on ER stress in CB13-treated NSCLC cells. Inhibition of PERK or CHOP blocks CB13-induced ER stress and apoptosis, indicating that CB13 causes cell death via ER stress. Excessive levels of ROS generation cause cellular damage via the activation of cell death such as apoptosis and ER stress. Recent studies suggest that multi-vesicular body (MVB) formation and exosomes increase during ER stress, and PERK and IRE1α deficiencies suppress ER stress-mediated exosome release^52^. We isolated exosomes from the growth medium of CB13-treated NSCLC cells in a time-dependent manner, and the levels of exosomal marker protein CD63 were higher in CB13-treated exosomes when compared to control cells. We observed up-regulation of GRP78 and CHOP during these exosome experiments, and exosomes isolated from PERK knock-down cells inhibited CB13-induced CD63 and CHOP levels, indicating an inhibition of ER stress. Thus, these result suggest new insight into how exosomes containing GRP78 regulate ER stress and cell death in CB13-treated tumor microenvironment.

Radio-resistance frequently manifests during tumor radiotherapy and is considered a major clinical problem^53^. Unfortunately, the underlying mechanism that causes radio-resistance and an effective tumor therapeutic strategy remain unclear^54^. To identify whether CB13 overcomes radio-resistance, radio-resistant A549R and H460R cells were established with continuous radiation exposure. The cell survival fraction data indicate that CB13 treatment causes a lower cell survival fraction in radio-resistant NSCLC cells when compared to control cells, suggesting that CB13 overcomes radio-resistance. Treating A549R and H460R cells with CB13 reduced cell viability and enhanced LDH release when compared to cells treated with 2 Gy. Treating radio-resistant NSCLC cells with a combination of 2 Gy and CB13 caused a synergistic decrease in cell viability and a greater increase in LDH cytotoxicity than in cells treated with 2 Gy or CB13, alone. We may expect that CB13 in combination with radiation exposure used effective clinical therapy for overcoming radiation resistance problem. To investigate the molecular mechanism used by CB13 to overcome radio-resistance, we examined ER stress pathways. Western blot analysis indicated that CB13, in combination with 2 Gy, induces ER stress and cell death by increasing the expression of p-PERK and CHOP, and the cleavage of caspase-3 in A549R and H460R cells when compared to cells treated with 2 Gy or CB13, alone. Together, CB13 and 2 Gy have a synergistic effect on ER stress and cell death in radio-resistant cells, which indicates 2 Gy/CB13 can overcome radio-resistance. In these circumstances, PPARɣ ligand-mediated ER stress could become an effective strategy to overcome radio-resistance. Our data suggest that CB13 regulates ER stress and apoptosis via activation of PPARɣ in NSCLC cells. We established PPARɣ knockdown-stable H460R cells using PPARɣ shRNA, and when treated with CB13, 2 Gy, or 2 Gy/CB13, they showed no decrease in cell viability; no up-regulation of PPARɣ, p-PERK, and CHOP expression; and no increase in caspase-3 cleavage. Hence, it identified that radiation/CB13 overcome radiation resistance via ER stress.

In conclusion, CB13 overcomes radio-resistance and induces cell death through PPARɣ activation, ROS generation, and ER stress in NSCLC and radio-resistant NSCLC cells; however, inhibition of ROS or PPARɣ blocks cell death in CB13-treated NSCLC and radio-resistant NSCLC cells. Our findings suggest that CB13 is a novel chemotherapeutic anti-cancer agent and potential drug that can overcome radio-resistance in NSCLC cells.

## Materials and methods

### Reagents

Propidium iodide (PI), and antioxidants including apocynin (Apo, a ROS inhibitor) and diphenyleneiodonium (DPI, a Nox inhibitor) were purchased from Sigma-Aldrich (St. Louis, MO). Cig and the selective PPARɣ antagonist GW9662 were obtained from Cayman (Ann Arbor, MI). The PPARɣ agonist candidate, CB13, was obtained from Chembridge (USA).

### Cell culture

Pre-adipose mouse cell lines (3T3-L1) and human non-small cell lung cancer (NSCLC) cell lines (A549 and H460) were purchased from the American Type Culture Collection (ATCC, Manassas, VA). Cells were grown in Dulbecco’s modified Eagle’s medium (DMEM) supplemented with 10% fetal bovine serum (FBS), 100 U/mL penicillin, and 100 μg/mL streptomycin (Gibco BRL Life Technologies, Gaithersburg, MD, USA) at 37 °C under 5% CO_2_. Stock solutions of Cig (10 mM) and CB13 (50 mM) were prepared in dimethyl sulfoxide (DMSO). Cig (10 μM) and CB13 (30 μM) were added to the culture media in the presence or absence of antioxidants. Culture medium containing 0.2% FBS was used when cells were treated with PPARγ ligands.

### Oil Red O staining

3T3-L1 preadipocytes were pretreated with Cig (10 μM), CB13 (30 μM), or the PPARɣ antagonist GW 9662 along with differentiation inducers. 3T3-L1 cells were seeded in a six-well plate at a density of 2 × 10^4^ cells per well in DMEM supplemented with 10% FBS, 100 U/mL penicillin, and 100 μg/mL streptomycin. After 3T3-L1 cells maintained 80% confluency for 2 days, the medium was exchanged with DMEM containing 10% FBS, 1 µM dexamethasone (Dex, Sigma-Aldrich), 0.5 mM 3-isobutyl-1-methyl-xanthine (IBMX, Sigma-Aldrich), and 5 µg/mL insulin (Sigma-Aldrich) for 48 h. After differentiation, the medium was exchanged with DMEM supplemented with 10% FBS and 5 µg/mL insulin twice every 48 h. At day 8, the ability of differentiated cells to accumulate intracellular lipids was assessed by Oil Red O staining. Cells were washed twice with PBS and chemically fixed with a 3.7% formaldehyde solution. The fixative solution was removed by rinsing twice with PBS, and the cells were stained with an Oil Red O working solution for 30 min at room temperature. The stain was removed by washing three times with deionized and distilled water. Cells were then visualized by phase-contrast microscopy (Olympus, Tokyo, Japan), and images were obtained with a digital camera (Camedia C-5060, Japan). The Oil Red O stain was then eluted with isopropyl alcohol, and its absorbance was measured at 510 nm.

### Cell viability

Cell viability was determined using a WST-1 (4-[3-(4-iodophenyl)-2-(4-nitrophenyl)-2H-5-tetrazolio]-1,3-benzene disulfonate) assay (Roche Applied Science, Indianapolis, IN, USA). Cell absorbance was measured at 450 nm using an enzyme-linked immunosorbent assay reader (SpectraMax190, Microplate Reader, Molecular Devices, CA, USA). The WST-1 assay used human NSCLC cell lines (A549 and H460) and radio-resistant NSCLC cell lines (A549R and H460R) plated in a 96-well plate overnight. The cells were treated with various concentrations of CB13 for various amounts of time.

### LDH assay

Human NSCLC cells were seeded and grown on a 96-well plate with growth medium. To determine the LDH (Thermo Scientific Pierce) activity in supernatants, an LDH assay was performed with 100 μl of the LDH reagent, and the solution was incubated for 30 min in a dark room. The LDH activity was determined by measuring the absorbance of the samples at 490 or 492 nm using an ELISA plate reader.

### Caspase-3 and caspase-9 activity assay

A caspase-3 and caspase-9 activity assay (the Biovision colorimetric caspase-3 and caspase-9 assay kit) was performed according to the manufacturer’s instructions. A549 and H460 cells were treated with Cig (30 μM; 24 h) and CB13 in a dose-dependent manner (0, 10, and 30 μM; 24 h). Cell lysis buffer (50 μl) was added to the cells and incubated for 10 min on ice. Samples were centrifuged at 10,000 × *g* for 1 min, and the protein concentration were quantified. Twenty (20) μg of total cellular protein was prepared and mixed with 2× reaction buffer (50 μl) and 4 mM DEVD-*p*NA substrate (5 μl) or 4 mM LEHD-*p*NA substrate (5 μl). After incubating for 1 h at 37 °C, the caspase-3 and caspase-9 activities were analyzed at 405 nm using a spectrophotometer (Molecular Devices).

### Colony formation assay

NSCLC and radio-resistant NSCLC cells were trypsinized, counted, and plated onto 60-mm dishes at a density of 1000 cells/dish. They were then treated as indicated and cultured for 10–12 days to allow for colony formation. The colonies were fixed and stained with 0.5% crystal violet in 50% ethanol, and dishes containing more than 100 cells per plate were counted. The survival fraction was calculated using the following formula: surviving fraction = number of colonies formed/number of cells seeded × plating efficiency of the control group.

### FACs

For cell cycle analysis, NSCLC cells were fixed with 80% cold ethanol, stained with a solution containing PI and RNase A (Sigma), and exposed to CB13 (30 μM) for 24 h. CB13-induced ROS generation during apoptosis was monitored by staining the cells with cell-permeant 2′7′-dichlorodihydrofluorescein diacetate (CM-H_2_DCFDA, Invitrogen). For Annexin V/PI measurements, the cells were stained with the dye for 30 min using the Annexin V-FITC kit (Biovision, Palo Alto, CA, USA) in accordance with the manufacturer’s instructions. The number of apoptotic cells was evaluated using the FACScan cytometer (Program Cell Quest, BD Biosciences).

### Irradiation

Ionizing radiation exposure was performed using ^137^Cs as the radiation source (Atomic Energy of Canada, Ltd., Mississuga, ON, Canada). For combination treatments, NSCLC and radio-resistant NSCLC cells were pre-treated with CB13 (30 μM) for 2 h, stimulated, and incubated for 24 h after 2, 4, or 6 Gy exposure.

### Generation of radio-resistant A549 and H460 cell lines

Cells were subjected to 2 Gy radiation daily for 3 months, taking weekends off. Throughout the irradiation process and recovery time, cells were kept at 40–70% confluency to ensure exponential growth. After completing the second week of radiation treatment, the cells were maintained in media containing 10% FBS. They were washed and re-fed daily for the first 7 days and then every third day thereafter. Radio-selected radio-resistance was verified by comparing the radio-sensitivity of the radiation-selected cells with their respective parental cell lines using a colony survival assay.

### Transfection

Human NSCLC cells in a six-well plate were transfected with double-stranded siRNAs (30 nmol/mL) against PERK (Santa Cruz), CHOP (Santa Cruz), and PPARɣ (Santa Cruz) and shRNAs against PPARɣ (Santa Cruz) for 24 h using Lipofectamine 2000 (Invitrogen, Grand Island, NY) according to the manufacturer’s protocol. For the luciferase reporter assay, plasmids were transiently transfected using Lipofectamine2000. To confirm luciferase reporter assay results, the luciferase pGL3 vector (2 µg, Promega) and the PPRE-luciferase pGL3 vector (2 µg, Promega) were co-transfected with 0.2 g of CMV-β-GAL, a eukaryotic expression vector in which the *Escherichia coli* β-galactosidase (Lac Z) structural gene is under the transcriptional control of the CMV promoter. Luciferase reporter activity was assessed on a luminometer with a luciferase assay system (Promega, Madison, WI) according to the manufacture’s protocol. The luciferase assay data represent the mean ± SD of three independent experiments.

### Western blot analyses

Human NSCLC cells were solubilized in radioimmunoprecipitation assay (RIPA) lysis buffer (Bio-rad). The primary antibodies used were: β-actin (Santa Cruz, 1:1000); CD63 (Abcam, 1:1000); PPARɣ (Proteintech, 1:1000); and cleaved PARP, cleaved caspase-3, cleaved caspase-9, p-PERK (Thr980), ATF4, CHOP, and p-eIF2ɑ (Ser51) (Cell Signaling, 1:1000). Primary antibodies were detected using a horseradish peroxidase-conjugated secondary antibody, and the membranes were visualized with Western Chemiluminescent HRP Substrate (Millipore).

### Measuring ROS

NSCLC cells were exposed to CB13 (30 μM) for 8 h. ROS generation was measured after staining with 5-(and-6)-carboxy-2′,7′-dichlorodihydrofluorescein diacetate (DCF-DA; Molecular Probes), which interacts with ROS to form a fluorescent complex. DCF fluorescence was immediately measured by FACS Calibur flow cytometry (Becton Dickinson). The data were acquired and analyzed using the Cell Quest Pro software.

### Exosome isolation

Exosomes were obtained from the cell culture supernatants of A549 and H460 cells treated with DMSO and CB13 (30 μΜ) using the total exosome isolation reagent (for cell culture media) according to the manufacturer’s protocol (Thermo Fisher Scientific). Protein concentrations were measured using the BCA method (Thermo Scientific). The protein samples (15 μg) were also quantified by Ponceau S staining. Positive exosomes were identified using the exosome marker, CD63.

### Statistical analysis

Data are expressed as the mean ± standard error (SE). Statistical analyses of the experimental data were performed using a two-sided Student’s *t*-test. *P-*values < 0.05 were deemed statistically significant.

## Supplementary information

Supplementary Figure 1

Supplementary Figure 2

Supplementary Figure 3

SUPPLEMENTAL MATERIAL
