# Supplementary figures and images for: CB13, a novel PPARγ ligand, overcomes radio-resistance via ROS generation and ER stress in human non-small cell lung cancer
Source: Cell Death Dis. 2020 Oct 13;11(10):848. doi: 10.1038/s41419-020-03065-w (PMC7555888; doi:10.1038/s41419-020-03065-w)

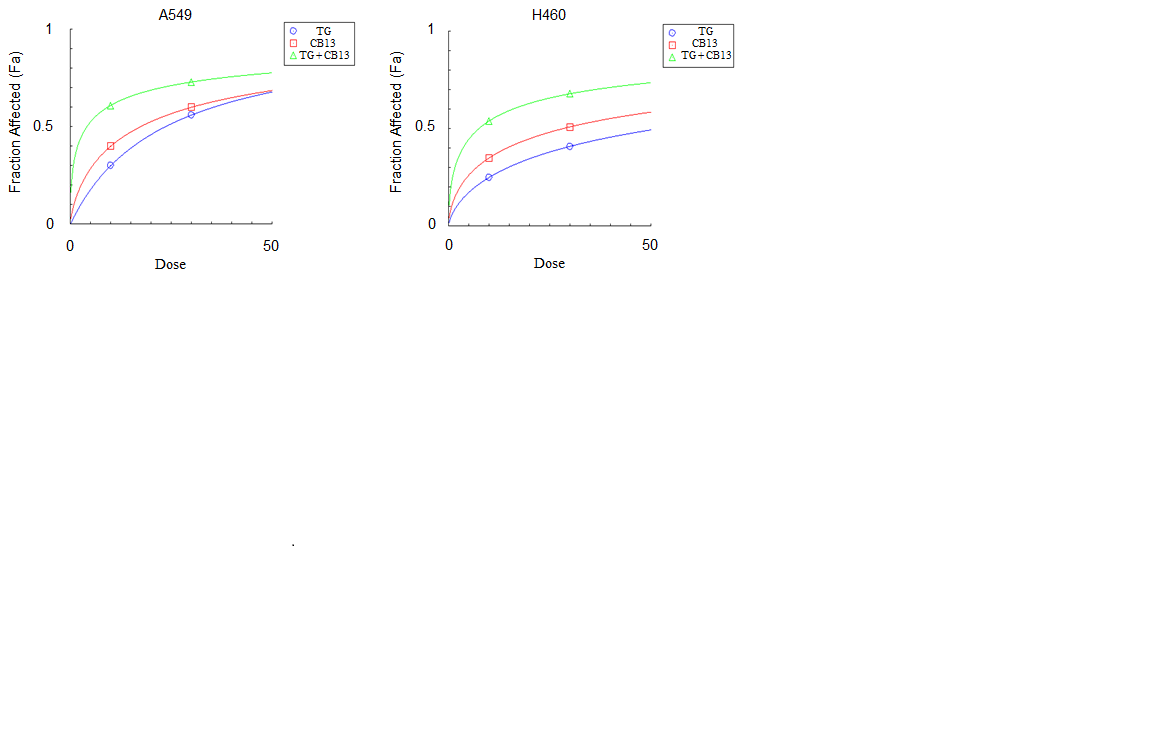

Supplement: Supplementary file 1 — Supplementary Figure 1 [file 41419_2020_3065_MOESM1_ESM.tif]

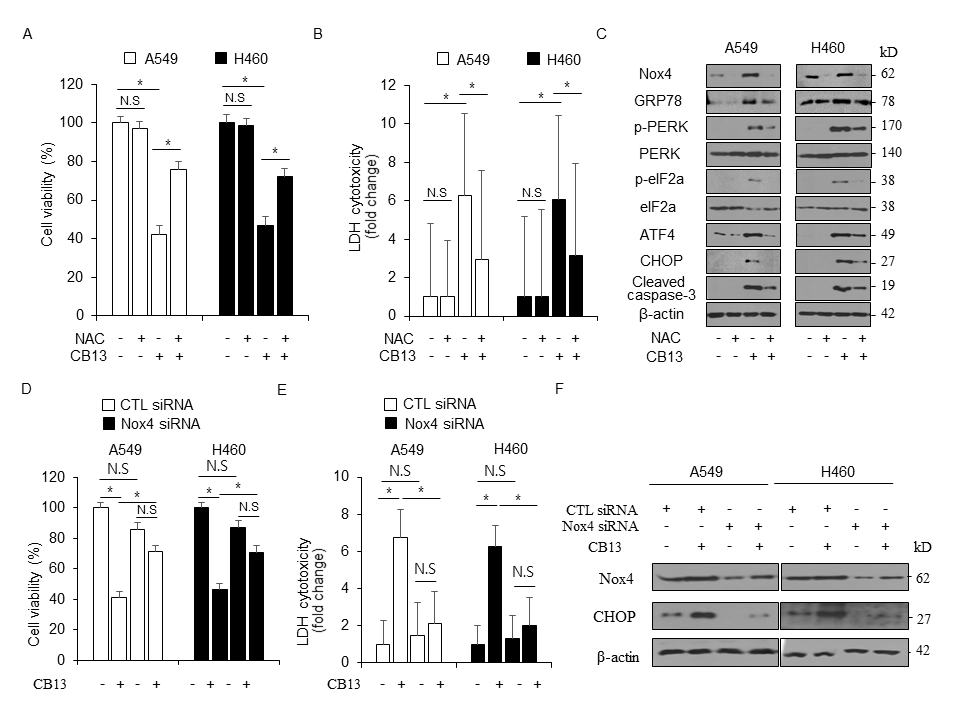

Supplement: Supplementary file 2 — Supplementary Figure 2 [file 41419_2020_3065_MOESM2_ESM.tif]

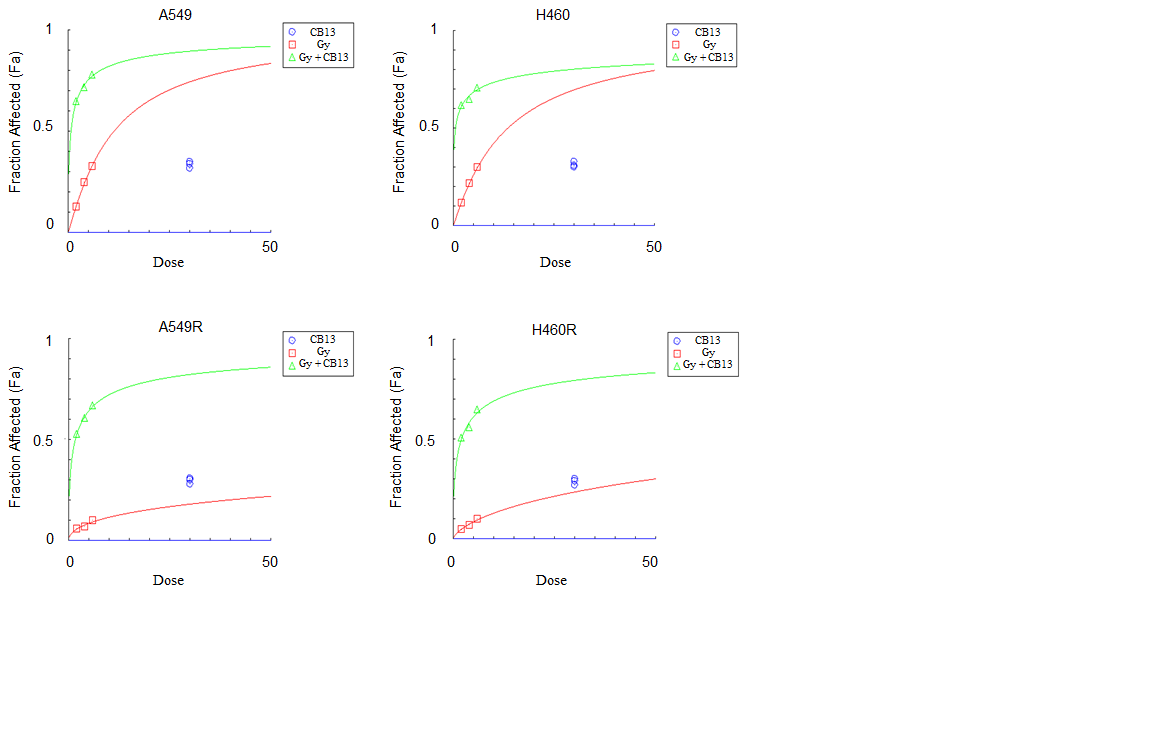

Supplement: Supplementary file 3 — Supplementary Figure 3 [file 41419_2020_3065_MOESM3_ESM.tif]
