# Supplementary material for: CB13, a novel PPARγ ligand, overcomes radio-resistance via ROS generation and ER stress in human non-small cell lung cancer
Source: Cell Death Dis. 2020 Oct 13;11(10):848. doi: 10.1038/s41419-020-03065-w (PMC7555888; doi:10.1038/s41419-020-03065-w)
Supplement: Supplementary file 4 — SUPPLEMENTAL MATERIAL [file 41419_2020_3065_MOESM4_ESM.docx]

**Supplementary Materials: CB13, a novel PPARγ ligand, overcomes radio-resistance via ROS generation and ER stress in human non-small cell lung cancer**

**Supplementary Figure Legends**

**Figure S1.**

**A mathematical isobologram analyses (dose-effect curves) obtained from the CompuSyn software for TG (1 and 3 μΜ, 24h) and CB13 (10 and 30 μΜ, 24h) combinations in A549 and H460 cells.**

**Figure S2.**

**NAC inhibits CB13-mediated apoptotic cell death in NSCLC cells..** (A-C) A549 and H460 cells were treated with NAC (2 mM, 24h) and CB13 (30 μΜ, 24h). Cell viability was determined using a WST-1 assay, and LDH cytotoxicity was measured using an LDH assay; *, P<0.05. A Western blot analysis examining Nox4, GRP78, p-PERK, p-eIF2ɑ, ATF4, CHOP, and cleaved caspase-3 levels was performed using these samples. β-actin was used as the protein loading control. (D-F**)** After A549 and H460 cells were transfected with Nox4 siRNAs, cell viability assays, LDH assays, and Western blot analyses were performed to examine Nox4 and CHOP levels after CB13 (30 μΜ, 24h) treatment; *, P<0.05. β-actin was used as the protein loading control.

**Figure S3.**

**A mathematical isobologram analyses (dose-effect curves) obtained from the CompuSyn software for Gy (2, 4 and 6 Gy, 24h) and CB13 (30 μΜ, 24h) combinations in A549, A549R, H460 and H460R cells.**
